# Genetic variants in mammary development, prolactin signalling and involution pathways explain considerable variation in bovine milk production and milk composition

**DOI:** 10.1186/1297-9686-46-29

**Published:** 2014-04-29

**Authors:** Lesley-Ann Raven, Benjamin G Cocks, Michael E Goddard, Jennie E Pryce, Ben J Hayes

**Affiliations:** 1 Biosciences Research Division, Department of Primary Industries Victoria, AgriBio, 5 Ring Road, Bundoora 3086, Australia; 2 La Trobe University, Bundoora, Victoria 3086, Australia; 3 Dairy Futures Co-operative Research Centre, Bundoora, Victoria 3086, Australia; 4 Faculty of Land and Food Resources, University of Melbourne, Parkville, Victoria 3010, Australia

## Abstract

**Background:**

The maintenance of lactation in mammals is the result of a balance between competing signals from mammary development, prolactin signalling and involution pathways. Dairy cattle are an interesting case study to investigate the effect of polymorphisms that affect the function of genes in these pathways. In dairy cattle, lactation yields and milk composition (for example protein percentage and fat percentage) are routinely recorded, and these vary greatly between individuals. In this study, we test 8058 single nucleotide polymorphisms in or close to genes in these pathways for association with milk production traits and determine the proportion of variance explained by each pathway, using data on 16 812 dairy cattle, including Holstein-Friesian and Jersey bulls and cows.

**Results:**

Single nucleotide polymorphisms close to genes in the mammary development, prolactin signalling and involution pathways were significantly associated with milk production traits. The involution pathway explained the largest proportion of genetic variation for production traits. The mammary development pathway also explained additional genetic variation for milk volume, fat percentage and protein percentage.

**Conclusions:**

Genetic variants in the involution pathway explained considerably more genetic variation in milk production traits than expected by chance. Many of the associations for single nucleotide polymorphisms in genes in this pathway have not been detected in conventional genome-wide association studies. The pathway approach used here allowed us to identify some novel candidates for further studies that will be aimed at refining the location of associated genomic regions and identifying polymorphisms contributing to variation in lactation volume and milk composition.

## Background

There have been many attempts to identify the genes that control milk production and functional traits in dairy cattle since they have high economic value [1,2]. Linkage studies and genome-wide association studies (GWAS) have led to the identification of a handful of causative mutations that affect milk production traits in dairy cattle [3-7]. However, the mutations that underlie most of the genetic variation remain elusive, reflecting the fact that the majority of these mutations are likely to have small effects and, therefore, individually explain a small proportion of the genetic variance [8,9]. New methods are needed to analyse the large quantity of genetic information provided by high-density SNP (single nucleotide polymorphism) panels in order to identify novel genetic variants that have a functional role in lactation traits.

One potential approach is to first filter genetic variants for association analysis by considering pathways of genes that are likely to be involved in lactation. The advantage of this method is that less stringent significance thresholds can be used than in traditional GWAS, since the level of multiple testing is not as high. This also means that associations of smaller effect can be detected. However, the approach does have the limitation that any mutations that affect the traits outside the selected pathways will be missed, which means that the variation we can identify may be reduced compared with that from whole-genome studies.

For dairy traits, genes that are involved in mammary gland development, prolactin signalling and involution pathways are relevant candidates. Genes in the lactation pathway have been well-described but are largely inferred from mouse studies [10-13]. Development of the mammary gland (or mammogenesis) involves the formation of the rudimentary mammary structure before puberty and is triggered by secreted signalling proteins and transcription factors that regulate developmental processes, such as the Wnt, notch and hedgehog signalling pathways [12]. When the mammary structure begins to form, genes for growth hormone and proteins involved in basement membrane architecture are expressed. At puberty, the concentration of several hormones increases and stimulates the formation of alveolar buds [14]. Prolactin signalling is vital for lobulo-alveolar development and establishment of lactation but appears less important after teat formation in dairy cattle [15,16]. One hypothesis is that in cattle, prolactin may be more important for immune support at calving [17]. Prolactin interacts with its receptors to trigger paracrine signalling mechanisms through a highly regulated feedback mechanism involving JAK/STAT and map kinase activity, as well as other downstream targets, which in turn regulate proliferation and cell differentiation [14]. In involution, milk producing epithelial cells are removed via cell detachment and apoptosis. Cytokines, interleukins and MMP (matrix metalloproteinases) are involved in complex signal transduction cascades to regulate proliferation and apoptosis in this pathway. The mammary epithelium undergoes several rounds of proliferation, differentiation and apoptosis over up to eight lactations in dairy cattle [18]. These processes are regulated by a number of genes, which represent excellent candidates for harbouring mutations that explain part of the observed variation in milk production traits and thus link genetic variation with the biological mechanisms underlying the phenotype. In this study, we have assembled sets of genes involved in mammary gland development, prolactin and involution biological pathways. Then, we tested SNPs in windows of 200 kb surrounding these genes for association with milk production traits in dairy cattle. Our hypothesis is that genes in these pathways will harbour genetic mutations that explain variation in production traits in dairy cattle, and that our approach will detect more of these associations than a traditional GWAS, since we can test variants at lower significance thresholds because of the smaller number of tests conducted.

## Methods

### Genome-wide association studies

To determine whether SNPs within key lactation pathways were significant for milk production traits, an association analysis was used. We analysed several traits, including fat kg, fat percentage, milk volume, protein kg, and protein percentage [19,20]. A total of 16 812 dairy cattle were genotyped using the Illumina Bovine HD BeadChip, or the BovineSNP50 array [21] and imputed to the higher density [22] (1785 animals were actually genotyped at the higher density). After quality control (as in [22]), the final number of SNPs was 632 003. The genotyped animals included 9015 Holstein cows, 2770 Holstein bulls, 4202 Jersey cows, and 825 Jersey bulls [see Additional file 1: Table S1]. Phenotypes of bulls and cows were constructed as daughter trait deviations (the average of the bull’s daughters trait deviations corrected for breed of mate) and trait deviations, respectively (corrected for herd year season and permanent environment effects) [see Additional file 2: Table S2]. The distributions of the number of lactations (for cows) and daughters (for bulls) are in Additional file 3: Figure S1. Records were standardised in both breeds to have a mean of 0 and a standard deviation of 1. In all analyses, phenotypes on bulls were weighted as

$$\frac{1-h^{2}}{\left[\frac{1+\left(4-h^{2}\right)}{n}\right]}$$

where *n* represents the number of records [23] and *h*^*2*^ is the heritability of the trait (0.33 for milk volume, fat kg and protein kg, and 0.5 for protein percentage and fat percentage, for both breeds [20]). Phenotypes on cows were weighted using the formula [23]:

$$\frac{1-h^{2}}{\left[\frac{1+r^{2}\left(l-1\right)}{l}\right]}$$

where *r*^2^ is the repeatability (0.56 for all milk production traits) and *l* is the number of lactations. For the percentage traits, we were not able to fit weights for bulls in the model due to problems with convergence, likely because the heritability for these traits was high.

The linear mixed model used to determine the association between individual SNP and each milk production trait:

y=Xβ+Wb+Zu+e

where **y** is the vector of phenotypes, expressed as the trait deviations for cows and daughter trait averages for bulls, **β** is the vector of fixed effects, including the overall mean and the effects of breed and sex, **X** is a design matrix allocating phenotypes to fixed effects, **W** is the vector of animal genotypes (the number of copies of the second allele at the SNP that the animal carries, coded as 0, 1 or 2), b is the additive effect of the second allele of the SNP, **Z** is an incidence matrix mapping phenotype to animals, **u** is the vector of polygenic effects (one for each animal), and **e** is the vector of random residuals. The polygenic breeding values were fitted as random effects following a normal distribution $N\left(0,A\sigma_{\alpha }^{2}\right)$ where **A** is the expected relationship among individuals constructed from the pedigree (which dates back to the 1940s) and $\sigma_{a}^{2}$ is the polygenic genetic variance. Variance components and fixed effects were estimated for each SNP with ASReml [24].

### Analysis of key lactation pathways

Gene sets for analysis were chosen using published reviews of three important developmental stages of the lactating mammary gland. These included the mammary development pathway [12] and the prolactin signalling [14] and involution pathways [25]. We identified 64 genes involved in mammary development, 27 genes involved in prolactin signalling, and 40 genes involved in involution (Tables 1, 2 and 3). The gene families *MAP kinase*, *P13K* and *frizzled* were not included in the pathways since specific genes were not suggested in the reviews and these gene families have a wide range of signalling functions. The genomic location of these genes were determined using UMD3.1 in the NCBI database [26]. The SNPs within the genes of a pathway, or within 100 kb to each side of those genes, were then tested for association with each trait using the model above. The effect of a SNP was determined to be significant at P ≤ 0.05. The GWAS was repeated using other significance thresholds (P < 10^−3^ and P < 10^−5^) but 0.05 had the greatest power to detect enrichment (results not shown). The number of SNPs significant for each pathway was expressed as a proportion of the total number of SNPs in that pathway (*PropSig*).

**Table 1:** Proportion of significant SNPs for genes in the mammary development pathway and number of SNPs significantly (P < 0.05) associated with each trait

**Gene**	**Chr**	**Start**	**Stop**	**Nb SNPs**	**Fat**	**Fat %**	**Milk**	**Protein**	**Prot %**
*ADAM17*	11	87798074	88040943	84	**0.51**	0.10	0.06	0.48	0.14
*AREGB*	6	91026256	91238391	53	0.42	**0.60**	**0.55**	**0.62**	**0.64**
*BMP4*	10	66651296	66855026	51	0.10	0.45	0.22	0.20	0.24
*BMPR1A*	28	41717915	41975988	55	0.00	0.02	0.20	0.31	0.04
*CSN2*	6	87079502	87288025	58	0.40	**0.59**	**0.83**	**0.78**	**0.90**
*CCND1*	29	47444380	47653820	43	0.35	0.35	0.16	0.12	0.44
*DKK1*	26	6752970	6955647	48	**0.50**	0.13	**0.58**	**0.58**	0.25
*EDAR*	11	44351547	44567795	31	0.26	0.13	0.23	0.39	0.23
*EGF*	6	16465618	16768065	103	0.08	0.09	0.12	0.12	0.00
*EGFR*	22	792005	1169280	81	0.23	0.05	0.11	0.16	0.05
*ESR1*	9	89869586	90355801	103	0.21	0.19	0.33	0.38	0.13
*FGF1*	7	55408007	55701836	65	**0.60**	0.15	0.11	**0.58**	**0.80**
*FGF10*	20	30510292	30719199	30	0.03	**0.50**	**0.50**	0.00	**0.63**
*FGFR1*	27	33150508	33400219	43	0.14	0.21	0.19	0.47	0.19
*GH1*	19	48668618	48872014	73	0.08	0.16	0.32	0.26	0.34
*GHR*	20	31790736	32299996	97	**0.62**	**0.93**	**0.84**	**0.54**	**0.96**
*GLI2*	2	72877209	73268370	82	0.16	0.10	0.11	0.27	0.05
*GLI3*	4	79344243	79858476	71	0.23	0.20	0.38	0.32	0.25
*IGF1*	5	66432877	66704734	49	0.37	0.24	0.31	0.24	0.18
*IGF1R*	21	8108822	8368093	61	0.07	0.05	0.11	0.10	0.07
*IRS1*	2	115690540	115894253	22	0.18	0.32	0.14	0.41	0.09
*IRS2*	12	88564525	88769181	64	0.28	0.02	**0.55**	0.28	**0.64**
*LEF1*	6	18235031	18550774	59	0.25	0.08	0.20	0.31	0.00
*MFGE8*	21	20789913	21004968	52	0.21	0.42	0.37	0.33	0.29
*MMP14*	10	21706054	21914533	43	0.19	0.02	0.12	0.19	0.00
*MMP2*	18	23728638	23955657	94	0.12	0.11	0.12	0.10	**0.55**
*MMP3*	15	5928011	6134595	52	0.06	0.23	0.15	0.13	0.02
*MMP9*	13	75366513	75573824	45	0.33	0.04	**0.56**	**0.53**	0.04
*MSX1*	6	105961463	106165759	56	0.43	**0.61**	0.46	0.38	**0.50**
*MSX2*	20	6260600	6465489	39	0.05	0.49	0.41	0.18	**0.54**
*NRG1*	27	27523938	27933470	79	0.18	0.24	0.29	0.30	0.15
*NRG3*	28	38201492	38451092	66	0.02	0.02	0.00	0.02	0.00
*NTN1*	19	28984419	29369338	87	0.36	0.23	0.37	0.34	0.31
*PGR*	15	8004485	8322755	64	0.48	0.13	0.38	0.48	0.06
*PCBD1/TCF1*	28	27126795	27331724	67	0.34	0.30	0.13	0.16	0.19
*PRL*	23	35005135	35213759	53	0.19	0.04	0.11	0.19	0.36
*PRLR*	20	38973246	39237480	56	0.48	**0.61**	**0.50**	0.25	**0.82**
*PTHLH*	5	82146522	82358858	30	0.30	**0.50**	0.47	0.33	0.33
*PTH1R*	22	53061302	53324114	48	0.40	0.15	0.13	0.35	0.23
*PTH*	15	39628332	39830868	31	0.23	0.06	**0.58**	0.19	0.26
*TNFRSF11A*	24	61139109	61375194	65	0.15	0.08	0.03	0.02	0.18
*TNFSF11*	12	12641069	12882474	73	0.33	**0.56**	0.05	0.08	**0.51**
*RELN*	4	44792394	45389293	131	**0.60**	0.07	**0.69**	0.34	0.47
*SIRPA*	13	53567570	53810792	80	**0.70**	0.09	0.46	**0.73**	0.48
*SLIT2*	6	41136589	41740789	145	0.09	0.18	0.17	0.19	0.46
*SOCS1*	25	9875299	10075970	56	0.30	0.00	0.16	0.32	0.00
*SOCS2*	5	23423981	23628860	46	0.28	**0.67**	**0.59**	**0.52**	**0.67**
*SOCS3*	19	54358856	54559555	62	0.44	0.21	0.18	0.40	**0.50**
*STAT5A*	19	42933597	43154075	59	0.36	**0.85**	**0.61**	**0.75**	**0.80**
*STAT5B*	19	42860226	43096671	60	0.10	**0.83**	**0.78**	**0.55**	**0.80**
*TBX2*	19	11843185	12051411	81	0.11	0.15	0.14	0.17	0.41
*TBX3*	17	62252245	62463636	45	0.27	0.20	0.33	0.18	0.29
*TCF3*	7	45499593	45730734	34	0.44	0.00	0.41	**0.50**	0.21
*TCF4*	24	54956409	55261459	73	0.03	0.14	0.19	0.23	0.05
*TGFA*	11	13772149	14086616	65	0.42	0.26	0.40	**0.55**	0.11
*TGFB1*	18	50671354	50885924	55	0.42	**0.58**	0.49	**0.71**	**0.65**
*TGFBR1*	8	64470093	64741796	64	0.23	0.33	0.33	0.27	0.41
*TGFBR2*	22	5041232	5333083	92	0.14	0.13	0.15	0.12	0.07
*WAP*	4	77111371	77312672	35	0.26	0.20	0.17	0.23	0.31
*WNT10B*	5	30913104	31114446	44	0.14	**0.91**	**0.91**	0.09	**0.86**
*WNT11*	15	56284700	56504335	51	0.37	0.35	0.22	0.31	0.12
*WNT3*	19	45921803	46171153	55	0.11	0.36	0.13	0.11	**0.78**
*WNT5A*	22	45996228	46212683	45	0.02	0.02	0.09	0.18	0.33
*WNT6*	2	107444683	107656681	64	0.34	**0.52**	**0.56**	0.44	0.34
**N = 64**				**3968**	**1079**	**1034**	**1247**	**1249**	**1350**

Bold values indicate where >50% of SNPs in a gene region were significant.

**Table 2:** Proportion of significant SNPs for genes in the prolactin pathway and number of SNPs significantly (P < 0.05) associated with each trait

**Gene**	**Chr**	**Start**	**Stop**	**Nb SNP**	**Fat**	**Fat %**	**Milk**	**Protein**	**Prot %**
*AKT2*	18	49804012	50050072	55	0.38	0.31	0.13	0.20	0.11
*CSN1S1*	6	87041556	87259096	98	0.07	0.18	0.05	0.09	0.22
*CSN2*	6	87079502	87288025	58	0.40	**0.59**	**0.83**	**0.78**	**0.90**
*CISH*	22	50220205	50425617	38	0.21	0.00	0.21	0.24	0.32
*RAF1*	22	57022412	57304951	115	0.34	0.06	0.47	**0.52**	0.01
*ELF5*	15	65724442	65954386	80	0.25	0.40	0.30	0.34	0.18
*ERBB4*	2	99560620	100097642	71	0.35	0.20	0.37	0.42	0.14
*ESR1*	9	89869586	90355801	103	0.21	0.19	0.33	0.38	0.13
*GAL*	29	46659818	46865617	59	0.15	0.29	0.07	0.31	0.37
*GATA3*	13	15884602	16102940	29	0.21	0.14	0.24	0.24	0.17
*IGF2*	29	49946626	50165230	27	0.37	0.04	0.30	0.22	0.15
*IL6*	4	31478311	31682667	25	0.40	0.00	0.24	0.36	0.00
*IRS1*	2	115690540	115894253	22	0.18	0.32	0.14	0.41	0.09
*JAK2*	8	39531342	39850796	32	0.28	0.47	**0.63**	**0.56**	0.44
*NR3C1*	7	56131970	56450496	65	**0.63**	0.37	0.28	**0.52**	0.43
*PRL*	23	35005135	35213759	53	0.19	0.04	0.11	0.19	0.36
*PRLR*	20	38973246	39237480	56	0.48	**0.61**	**0.50**	0.25	**0.82**
*PTH*	15	39628332	39830868	31	0.23	0.06	**0.58**	0.19	0.26
*SOCS1*	25	9875299	10075970	56	0.30	0.00	0.16	0.32	0.00
*SOCS2*	5	23423981	23628860	46	0.28	**0.67**	**0.59**	**0.52**	**0.67**
*SOCS3*	19	54358856	54559555	62	0.44	0.21	0.18	0.40	**0.50**
*GH1*	19	48668618	48872014	73	0.08	0.16	0.32	0.26	0.34
*STAT3*	19	42956660	43232624	58	0.43	**0.84**	**0.53**	**0.78**	**0.84**
*STAT5A*	19	42933597	43154075	59	0.36	**0.85**	**0.61**	**0.75**	**0.80**
*STAT5B*	19	42860226	43096671	60	0.10	**0.83**	**0.78**	**0.55**	**0.80**
*TNFRSF11A*	24	61139109	61375194	65	0.15	0.08	0.03	0.02	0.18
*TNFSF11*	12	12641069	12882474	73	0.33	**0.56**	0.05	0.08	**0.51**
**N = 27**				**1569**	**458**	**519**	**571**	**636**	**628**

Bold values indicate where >50% of SNPs in a gene region were significant.

**Table 3:** Proportion of significant SNPs for genes in the involution pathway and number of SNPs significantly (P < 0.05) associated with each trait

**Gene**	**Chr**	**Start**	**Stop**	**Nb SNP**	**Fat**	**Fat %**	**Milk**	**Protein**	**Prot %**
*AKT1*	21	70778138	70995537	30	0.17	0.17	0.13	0.13	0.17
*ATF4*	5	111362845	111564936	52	**0.54**	0.12	**0.52**	0.23	**0.60**
*BAK1*	23	7555892	7758885	31	0.19	0.16	0.23	0.03	0.45
*BAX*	18	55885202	56089378	35	0.11	0.49	**0.51**	0.40	0.49
*BCL2L1*	13	61666806	61917383	28	0.04	0.07	**0.50**	0.36	0.11
*CASP3*	27	13984622	14210610	49	0.12	0.10	0.16	0.18	0.08
*CEBPA*	18	43828610	44029840	30	0.10	0.13	0.20	0.07	0.13
*CEBPD*	14	20638814	20840407	28	0.46	0.25	0.46	0.43	0.32
*CEBPG*	18	43905707	44112657	68	0.24	0.06	0.15	0.16	0.03
*CISH*	22	50220205	50425617	38	0.21	0.00	0.21	0.24	0.32
*CTNNA1*	7	51588098	51980519	14	0.14	**0.79**	0.14	0.21	0.00
*CTNNA2*	11	54622279	56182035	514	0.23	0.40	**0.53**	0.48	0.38
*E2F1*	13	63605710	63814008	21	**0.62**	0.10	0.48	0.43	0.19
*FOXO3*	9	41908606	42218673	56	0.09	0.02	0.09	0.14	0.00
*IGFBP5*	2	105278991	105497646	61	**0.62**	0.08	0.21	**0.64**	**0.72**
*IL11*	18	62461915	62664977	61	**0.51**	0.11	0.05	0.36	0.16
*IL6*	4	31478311	31682667	25	0.40	0.00	0.24	0.36	0.00
*IL6ST*	20	23112633	23370316	64	0.23	0.39	0.28	0.39	**0.67**
*IRF1*	7	23135653	23343697	46	**0.72**	0.00	**0.61**	**0.67**	0.20
*JAK1*	3	80675557	81015026	65	0.18	0.45	**0.55**	0.35	0.46
*JAK2*	8	39531342	39850796	32	0.28	0.47	**0.63**	**0.56**	0.44
*LEF1*	6	18235031	18550774	59	0.25	0.08	0.20	0.31	0.00
*LIF*	17	71313855	71518166	53	0.13	0.02	0.09	0.26	0.08
*LIFR*	20	35817479	36066671	74	**0.54**	**0.58**	**0.68**	**0.58**	**0.80**
*MMP2*	18	23728638	23955657	94	0.12	0.11	0.12	0.10	**0.55**
*MMP3*	15	5928011	6134595	52	0.06	0.23	0.15	0.13	0.02
*MMP9*	13	75366513	75573824	45	0.33	0.04	**0.56**	**0.53**	0.04
*MYC*	14	13669244	13874438	38	**0.61**	**0.76**	0.24	0.13	0.32
*OSM*	17	71334468	71537372	51	0.16	0.06	0.06	0.24	0.08
*OSMR*	20	35421410	35688186	53	**0.60**	**0.70**	**0.64**	**0.58**	**0.74**
*TP53*	19	27885495	28097841	23	0.43	0.48	0.22	0.43	0.17
*PTEN*	26	9398226	9695849	33	0.03	0.36	0.42	0.36	0.42
*PTK2*	14	3770893	4165010	121	**0.79**	**0.90**	**0.84**	**0.66**	**0.82**
*RAF1*	22	57022412	57304951	115	0.34	0.06	0.47	**0.52**	0.01
*SFRP4*	4	49909882	50120466	51	0.22	0.49	**0.59**	**0.57**	0.47
*SOCS3*	19	54358856	54559555	62	0.44	0.21	0.18	0.40	**0.50**
*STAT3*	19	42956660	43232624	58	0.43	**0.84**	**0.53**	**0.78**	**0.84**
*STAT5A*	19	42933597	43154075	59	0.36	**0.85**	**0.61**	**0.75**	**0.80**
*STAT5B*	19	42860226	43096671	60	0.10	**0.83**	**0.78**	**0.55**	**0.80**
*TIMP3*	5	71651415	71909052	72	**0.54**	0.21	0.49	0.38	0.42
**N = 40**				**2521**	**803**	**841**	**1048**	**1044**	**972**

Bold values indicate where >50% of SNPs in a gene region were significant.

To determine if the proportion of significant SNPs observed for each pathway was significantly greater than by chance at an experiment-wise level, distributions under the null hypothesis of no association were constructed with random permutations of the data. A list of 24 617 uniquely annotated bovine genes was created from the Ensembl Biomart database [27,28]. From this, three sets of genes, each with a length equal to the respective pathway tested were selected at random. SNPs were selected from within and 100 kb surrounding these genes to reflect the moderate to high linkage disequilibrium in Holstein cattle [29,30]. Each pathway SNP set was analysed in ASReml using the mixed linear model described above. This procedure was repeated 10 000 times to construct null distributions and the 500th highest proportion of significant SNPs was taken at the experiment-wise P < 0.05 threshold. If the observed ratio for a pathway was greater than this value for a particular trait, the pathway was considered significant.

To account for differences in functional clustering of genes in the experimental pathways and in the random control gene sets, we compared the distance between genes on the same chromosome [see Additional file 4: Figure S2]. The experimental and control sets were distributed similarly but, due to the smaller number or paired genes for the experimental pathways, there were fewer gene pairs at long distances across the chromosomes (particularly > 10 Mb).

KEGG annotations were used to determine the gene sets that represented other biological pathways [31,32].

Finally, a variance component analysis was used to determine whether the SNPs within each pathway explained a greater proportion of the genetic variance than an equal number of randomly selected SNPs from the whole genome. The model fitted was

y=Wb+Zg+e,

where terms were the same as above, and **g** is a vector of random effects, assumed distributed $N\left(0,G\sigma_{g}^{2}\right)$, where **G** is a genomic relationship matrix, constructed using the rules of [33]. The genomic relationship matrix was based on the SNPs from each pathway, plus a set of 4000 SNPs randomly selected from the whole genome. The reason for adding the 4000 randomly chosen SNPs was that SNPs in the genes of the pathways are typically clustered by genomic location (i.e. a number of the genes are located in close proximity) [see Additional file 4: Figure S2]. Given the large number of animals in our dataset, this means that a considerable number of animals can have genomic relationships that are equal to or close to 1, i.e. they have inherited the same segments of the genome at all of the locations of the pathway genes. Consequently, the genomic relationship matrix is singular and impossible to invert. Adding 4000 random SNPs removed the singularities and the genomic relationship matrix could be inverted and variance components estimated. However, with the 4000 SNPs included, we could only assess the marginal contribution of adding SNPs in the pathway.

Estimates of the variance components $\sigma_{g}^{2}$ and $\sigma_{e}^{2}$ were obtained from the REML analysis with ASREML [24]. The proportion of variance explained by the SNPs in these pathways was compared to that explained by the same number of randomly chosen SNPs within 100 kb of a gene, i.e. the additional SNPs were chosen to be close to genes, plus the set of 4000 randomly chosen SNPs corresponding to each pathway. Five replicates of the randomly chosen sets were performed to obtain standard errors.

## Results

### Mammary development pathway

The 64 genes identified in the mammary development pathway included 3968 SNPs (Table 1). When the proportion of significant SNPs, at P < 0.05, (*PropSig*) was compared to the null distributions, the mammary development pathway was significantly associated with protein percentage (*PropSig* = 0.340, P < 0.01; Table 4 and Additional file 5: Figure S3). The null distributions compared with the experimental results are shown in Additional file 5: Figure S3, Additional file 6: Figure S4 and Additional file 7: Figure S5. The genes that contained the largest proportion of significant SNPs (> 50% significant SNPs) were the following: *AREGB*, *CASB*, *DKK1*, *FGF1*, *FGF10*, *GHR*, *PRLR*, *SOCS2*, *STAT5A*, *STAT5B*, *TGFB1* and *WNT10B* (Table 1 and Additional file 8: Table S3 for gene abbreviations).

**Table 4:** Proportion of significant SNPs for milk production traits in the mammary development, prolactin and involution pathway genes

	**Mammary development**		**Prolactin Signalling**		**Involution**	
**Trait**	***PropSig***	**μ( *PropSig *)**	***PropSig***	**μ( *PropSig *)**	***PropSig***	**μ( *PropSig *)**
Fat	0.272	0.266	0.292	0.267	0.319	0.267
Milk	0.314	0.295	0.364	0.296	**0.415	0.295
Protein	0.315	0.314	*0.405	0.315	**0.414	0.315
Fat %	0.261	0.221	**0.331	0.222	**0.333	0.221
Protein %	**0.340	0.262	*0.400	0.262	**0.385	0.262
**Nb SNP**	**3968**		**1569**		**2521**	

The observed proportion of SNPs significant was compared to the mean (μ) of the simulated null distribution (μ(*PropSig*)), created by permutation testing, to determine if the observed proportion was significant at an experiment-wise level (* denotes significant at P < 0.05 experiment-wise, ** denotes significant at P < 0.01 experiment-wise) (see Methods). The total number of SNPs is presented in bold.

Four genes in the mammary development pathway were located on BTA20, which contains a well-known QTL for milk production [5]. These genes included *FGF10*, *MSX2*, *PRLR* and *GHR. FGF10* is located 1 Mb downstream of *GHR*, which is the gene often described with, though not necessarily underlying [34], this large QTL. To account for any potential bias associated with over-represented genes, we re-ran the pathway test and control permutations without BTA20. The mammary development pathway still reached significance for protein percentage when this chromosome was removed [see Additional file 9: Figure S6].

KEGG annotations of these 64 genes found 25 genes in pathways associated with cancer and 8 to 14 other genes in signalling pathways, such as *JAK-STAT*, that are known to be activated during lactation (Table 5). The PI3K-Akt pathway is involved in mammary development, and mutations in genes of this pathway are found in approximately 70% of breast cancers [35]. There were eight genes involved in Wnt signalling pathways, which are prominent in mammary development and cancers [36].

**Table 5:** KEGG associations for the mammary development, prolactin signalling and involution pathways

**Mammary development**		
**ID**	**Pathway**	**Nb Genes**
bta05200	Pathways in cancer	25
bta05166	HTLV-I infection	14
bta04151	PI3K-Akt signalling pathway	14
bta04060	Cytokine-cytokine receptor interaction	12
bta04630	Jak-STAT signalling pathway	10
bta05217	Basal cell carcinoma	9
bta04380	Osteoclast differentiation	8
bta05218	Melanoma	8
bta04310	Wnt signalling pathway	8
bta04010	MAPK signalling pathway	8
**Prolactin signalling**		
**ID**	**Pathway**	**Nb Genes**
bta04630	Jak-STAT signalling pathway	12
bta04151	PI3K-Akt signalling pathway	8
bta04910	Insulin signalling pathway	6
bta04380	Osteoclast differentiation	5
bta05200	Pathways in cancer	5
bta05161	Hepatitis B	5
bta05164	Influenza A	5
bta04920	Adipocytokine signalling pathway	5
bta05162	Measles	5
bta04060	Cytokine-cytokine receptor interaction	5
**Involution**		
**ID**	**Pathway**	**Nb Genes**
bta05200	Pathways in cancer	18
bta04630	Jak-STAT signalling pathway	15
bta05161	Hepatitis B	14
bta04151	PI3K-Akt signalling pathway	13
bta05166	HTLV-I infection	9
bta05203	Viral carcinogenesis	9
bta05152	Tuberculosis	8
bta05213	Endometrial cancer	8
bta05210	Colorectal cancer	7
bta05202	Transcriptional misregulation in cancer	7

To determine the extent of pleiotropy for variants in the pathway, we correlated the SNP effect estimates (for the 3968 SNPs in the pathway) for each pair of traits. Milk volume was negatively correlated with fat percentage and protein percentage, while fat percentage and protein percentage were highly positively correlated (Table 6). Fat kg and milk volume were also highly positively correlated with protein kg, as expected.

**Table 6:** Correlation between core traits for SNP within the mammary development, prolactin signalling and involution pathways

**Mammary development**				
	**Fat**	**Milk**	**Protein**	**Fat %**
Milk	0.490			
Protein	**0.753**	*0.703*		
Fat %	0.221	*-0.719*	-0.178	
Protein %	0.121	*-0.618*	0.116	**0.792**
**Prolactin signalling**				
	**Fat**	**Milk**	**Protein**	**Fat %**
Milk	*0.524*			
Protein	*0.695*	*0.663*		
Fat %	0.140	*-0.748*	-0.227	
Protein %	0.145	-0.456	0.357	*0.643*
**Involution**				
	**Fat**	**Milk**	**Protein**	**Fat %**
Milk	0.097			
Protein	0.364	**0.825**		
Fat %	0.442	**-0.833**	*-0.539*	
Protein %	0.225	**-0.774**	-0.288	**0.806**

Bold values represent a high correlation, italicised values represent a moderate correlation and all other values correspond to low to zero correlation.

### Prolactin signalling pathway

The prolactin signalling gene set was considerably smaller (27 genes, 1569 SNPs) than the involution and mammary development sets, since it only represents only one signalling pathway, while mammary development and involution represent the combined effects of several sub-pathways (Table 2). Protein kg, fat kg and fat percentage were significantly associated with the prolactin signalling gene set (Table 4) and [see Additional file 6: Figure S4]. The *SOCS2*, *STAT3*, *STAT5A*, *STAT5B*, *PRLR* and *CASB* genes had more than 50% of SNPs significant for three or more milk production traits (Table 2).

KEGG annotations for genes in the prolactin pathway showed 12 associations with the JAK-STAT signalling pathway, followed by the PI3K-Akt and insulin signalling pathways (Table 5).

### Involution pathway

The involution pathway contained 40 genes and 2521 SNPs (Table 3). The proportion of associated SNPs was significant at the experiment-wise level for all milk production traits, except fat [see Additional file 7: Figure S5] and (Table 4). We identified a large ratio of significant SNPs for *ATF4*, *IGFBP4*, *IRF1*, *LIFR*, *OSMR*, *PTK2*, *STAT3*, *STAT5A* and *STAT5B* (Table 3). KEGG analysis showed a trend towards infection-related pathways (Table 5). JAK-STAT, hepatitis B and PI3K signalling pathways were also highly represented. Traits showed moderate to high correlations, which suggested pleiotropy for milk production traits within SNPs in the involution pathway (Table 6).

Three genes in the involution pathway were located on BTA14 and may be biased by associations with the large QTL at the beginning of BTA14 associated with the mutation in *DGAT1*[37]. The *CEPBD* and *MYC* genes are located more than 13 Mb upstream of this QTL but *PTK2* sits 2 Mb upstream from *DGAT1*, well within the bounds of this very large QTL. When BTA14 was removed from the analysis, the involution pathway remained significant for the traits for which this was tested [see Additional file 9: Figure S6].

**Figure 1:**
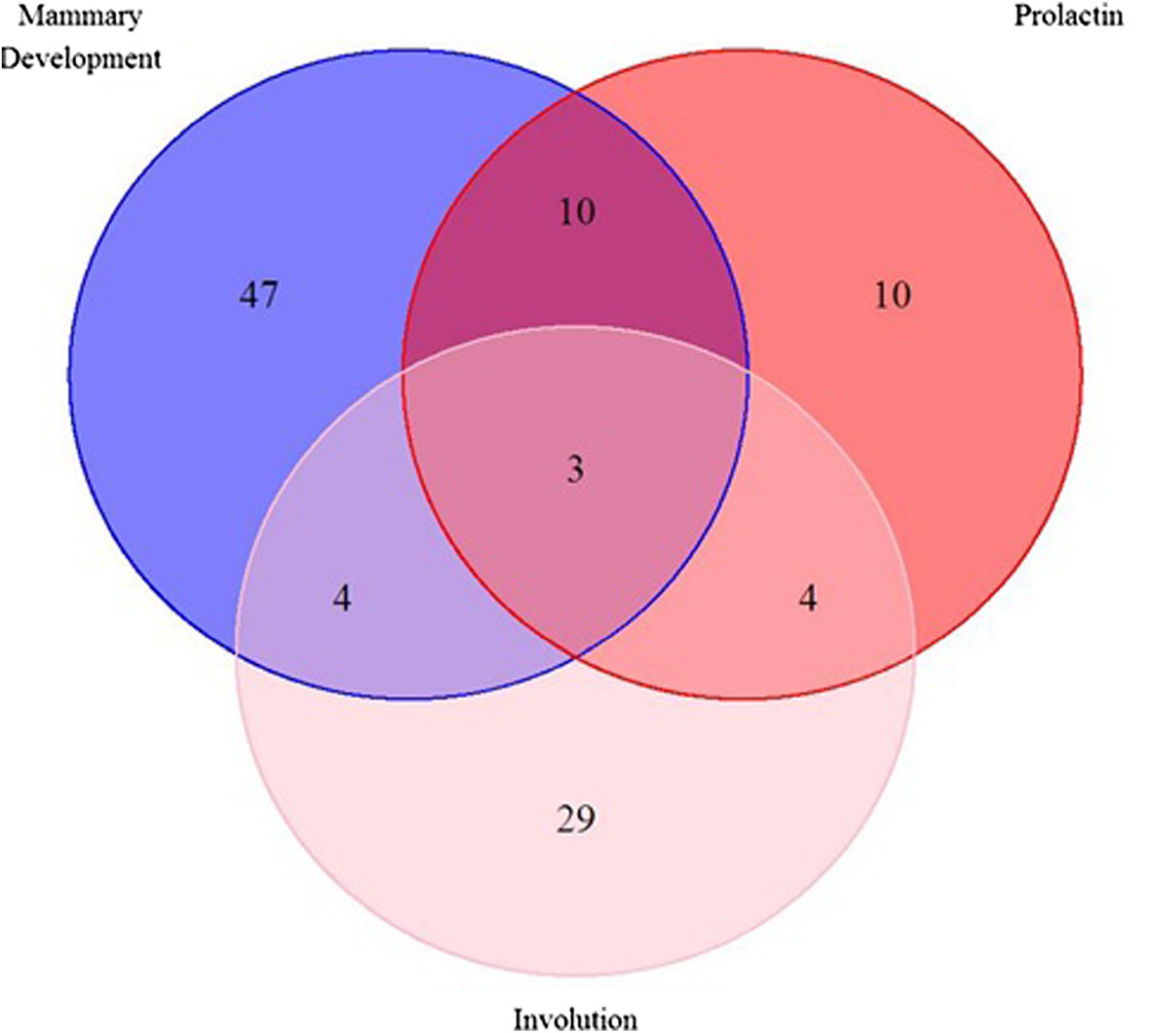
Venn diagram showing the number of overlapping genes in three lactation pathways.

There was some overlap in the genes of the three pathways. Genes *STAT5A*, *STAT5B* and *SOCS3* were common to all three pathways (Figure 1). Prolactin and mammary development pathways showed the largest overlap, which included *TNF*, *SOCS* and *prolactin* genes. KEGG analyses showed that similar pathways were represented in mammary development and involution but infection-related pathways were more prominent due to the abundance of acute phase response genes such as *interleukins* and *STAT* genes (Table 5).

### Proportion of variance explained by mutations in pathways

For milk production traits, SNPs in the involution pathway explained 10 to 13% more genetic variation than expected by chance for all traits (Table 7). SNPs in the mammary development pathway explained 7 to 9% more genetic variation than expected by chance for milk, protein percentage and fat percentage. SNPs in the prolactin pathway explained less variation than expected by chance, although results were not significantly different from zero. This could be the result of a combination of two factors, i.e. (1) SNPs within the prolactin signalling pathway do not really explain much variation, and (2) because of the small number of genes in this pathway, the SNPs did not cover all chromosomes (and therefore did not capture variation on those chromosomes), unlike the randomly sampled SNPs. The overall significance of each milk production trait for each pathway tested was very similar, though not identical, to the results from SNP by SNP association testing (perhaps a result of random sampling to construct the null distributions).

**Table 7:** Additional genetic variance explained by SNPs in genes or within 100 kb of genes in the mammary development, prolactin signalling, and involution pathways, compared with an equal number of randomly chosen SNPs within 100 kb of genes

	**Mammary development**	**Prolactin signalling**	**Involution**
Milk volume	**7.0 ± 3.2**	−1.3 ± 1.9	**13.3 ± 1.9**
Fat kg	2.0 ± 2.7	−1.2 ± 1.7	**11.0 ± 1.5**
Protein kg	−0.4 ± 3.5	−2.4 ± 2.0	**11.6 ± 2.8**
Fat %	**9.3 ± 3.2**	−0.3 ± 1.0	**12.1 ± 1.5**
Protein %	**6.8 ± 3.2**	−2.2 ± 1.4	**13.0 ± 2.3**

Standard errors were derived from the genetic variation explained in five random sets of SNPs; significant (based on the variance explained being greater than 2 standard errors) trait x pathway combinations are in bold.

## Discussion

We used information on mammary development, prolactin signalling and involution pathways to identify candidate gene regions that could be associated with milk production traits. SNPs in genes that are involved in the mammary development pathway were highly associated with protein percentage and explained a considerable proportion of the variance for three milk production traits. The prolactin signalling pathway did not explain any additional variance in milk production traits, but contained a significant number of associated SNPs for protein kg, protein percentage and fat percentage. SNPs in genes involved in the involution pathway explained the greatest level of variance in milk production traits in our variance component approach. The involution pathway was also significant for all milk production traits except fat in the association testing approach.

**Figure 2:**
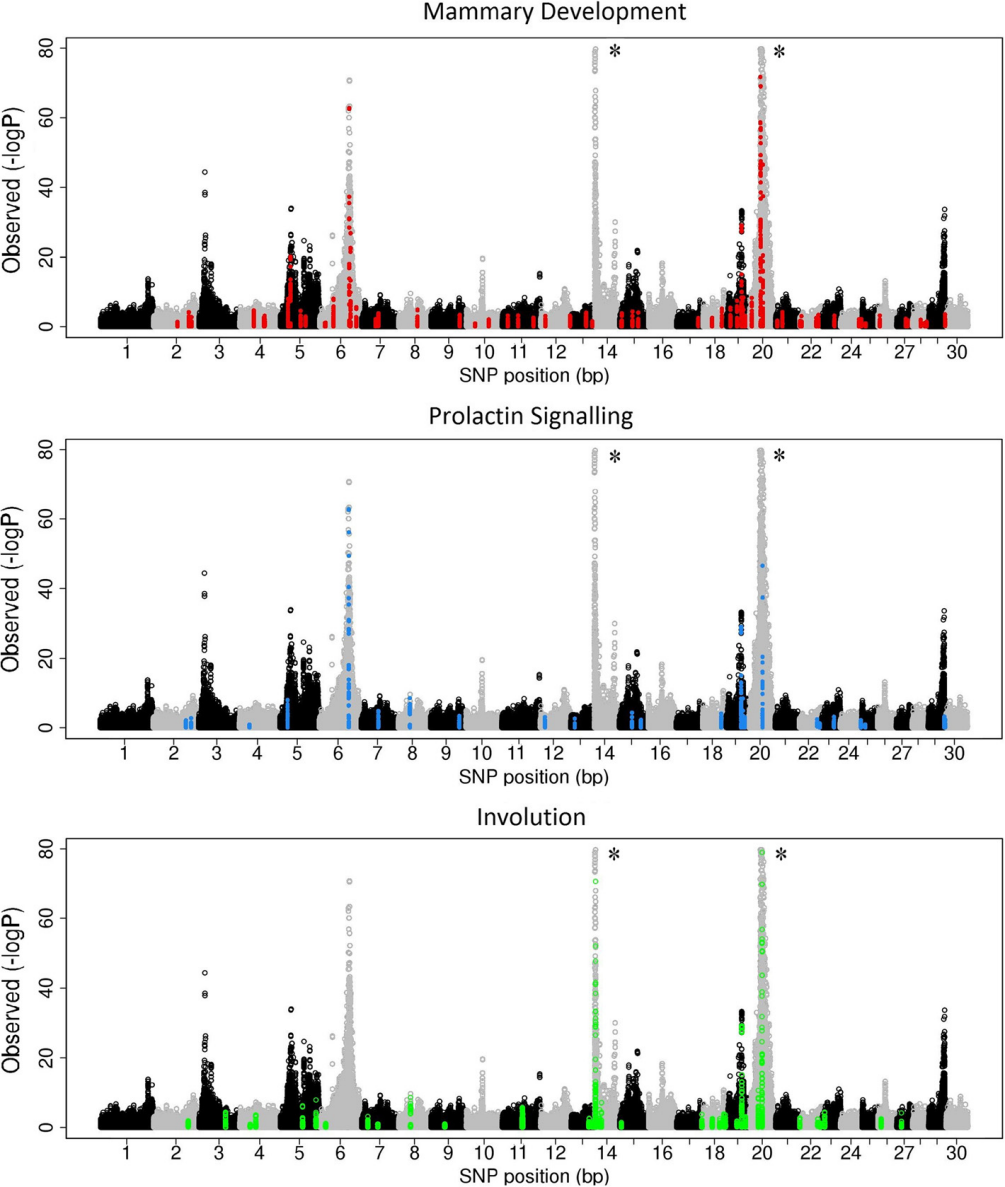
**GWAS of protein percentage in Holsteins and Jerseys.** SNPs within the mammary development, prolactin signalling, and involution pathways are highlighted as red, blue and green dots, respectively; * identifies chromosomes 14 and 20, which have been scaled down to allow observation of smaller effects.

Mammary development, prolactin signalling and involution pathways contained highly significant genes that have been described in GWAS or are known to be important lactation genes. These include, *CASB*, *SOCS2*, *GHR*, *PRLR*, *LIFR* and the *STAT* genes. In particular, SNPs within *STAT5A* have a large effect on milk composition and have been validated *in vitro*[38,39]. Figure 2 shows a GWAS for protein percentage as an example, and displays the relationship between genes studied from these pathways and genome-wide QTL patterns. Most genes are located in regions that could not be identified by a traditional GWAS. SNPs within regions not previously associated with milk production traits, such as *AREGB*, *ATF4*, *IRF1*, *DKK1*, and *TGFB1*, which were significant for mammary development, may contain novel mutations that affect milk production traits and may represent key genes from the mammary development pathway that explain some of the variance in these traits in cattle.

The reason why the involution pathway explained the greatest level of variance in milk production traits in our variance component approach, although only half the number of SNPs of the mammary development pathway were available, could be because this pathway includes genes in or close to a previously described QTL with quite large effects on milk production traits (Figure 2), particularly protein percentage [5]. However, when the analysis was ran without the genes on BTA20 (*FGF10*, *MSX2*, *PRLR* and *GHR*), this pathway was still significant, even for protein percentage. Note that removing the *GHR* gene from the analysis is questionable because the growth hormone receptor is a vital component of the lactation pathway since it interacts with several relevant substrates during lactation [5]. Similarly, removing the *CEBPD*, *MYC* and *PTK2* genes on BTA14 (because they were in the region of *DGAT1*) did not affect the overall significance of the mammary development pathway. The clustered expression of the genes in a pathway, i.e. they are expressed with other secreted milk genes [40], may result in significant associations that are due to nearby, co-expressed genes. The permutation method generated some replicates with similar genome distributions to the experimental data [see Additional file 4: Figure S2], which implies that the clustered expression of genes probably does not greatly affect the results. There is currently no ideal approach to control for the complicated genetic architectures of traits in pathway analyses. While these genetic structures should be accounted for, caution should be taken to avoid losing information from highly relevant genes.

One of the main limitations of our approach is that if a mutation that affects milk production is not in the analysed pathways, it will automatically be excluded. Perhaps even more importantly, our interpretations could be biased if irrelevant genes are included in the pathways. This may have occurred in cases where broad-acting cellular processes are represented in the gene sets. Improved descriptions of pathways would increase the power to identify genomic regions that influence these traits. The pathways used in this study were primarily derived from mouse studies and are relatively poorly described in cattle. For mammary development, the signalling interactions in the placode epithelium are particularly poorly described. For the prolactin signalling pathway, little is known about the downstream signalling of progesterone receptors. For the involution pathway, it is not known how membrane apoptosis is triggered although this would represent a significant contribution to the description of this biological process. Approaches such as microarray and RNAseq technologies using time-course data could help refine this method so that it represents more closely the true biological action. These approaches have successfully identified genes acting at different physiological states in the lactation cycle. Another potential limitation of our study is that the phenotypes were averages of several records across lactation. The same analyses could be performed using just early or late lactation records. Lactation curve parameters have been used in similar modelling experiments and may further refine these numerous SNP associations [41].

Finally, the value of KEGG pathway annotations was questionable. The relevance of these annotations for the target traits is difficult to establish for genes that are involved in broad and numerous biological processes. A further problem is that KEGG annotations are heavily dominated by cancer-related information.

## Conclusions

We have successfully used the information from characterised mammary development, prolactin signalling and involution pathways to identify novel SNP associations with milk production traits. The proportion of significant SNPs in or near genes from the mammary development pathway was considerably greater than expected by chance for protein percentage. Of the three pathways studied, the involution pathway was highly associated with milk production traits and explained the highest level of variation above that expected by chance (up to 13% for protein kg). While we have reported many novel candidates useful for further studies, we must point out that pathway-based methods are restricted by the quality of annotations and completeness of pathway information.

## Competing interests

The authors declare that they have no competing interests.

## Authors’ contributions

LR performed the GWAS and pathway analyses and drafted the manuscript. BC, BH, LR and MG conceived and designed the study. BH performed the variance component analysis. JP assisted in the interpretation. MG assisted with the pathway design. All authors read and approved the final manuscript.

## Supplementary Material

Additional file 1: Table S1a Number of phenotypes for production traits. Sample sizes adapted from [42]. **Table S1b.** The minimum and maximum phenotypes for production traits in dairy cattle. Phenotypes are expressed in standard deviations, with a mean of zero within each breed. Adapted from [42]. Click here for file

Additional file 2: Table S2 Description and measurement of milk production traits. All non-production traits are expressed as a percentage of the standard deviation from the phenotypic mean. Click here for file

Additional file 3: Figure S1a Distribution of number of lactations for cows. X-axis is labelled with the mid-point of each bin. **Figure S1b.** Distribution of number of daughters per bull. X-axis is labelled with the mid-point of each bin. Click here for file

Additional file 4: Figure S2 Distance between genes in lactation pathways and control permutations. Control plots are scaled to represent 10 000 replicates of randomly selected genes of size equivalent to the experimental pathway. Click here for file

Additional file 5: Figure S3 Permutation tests for SNP within the mammary development pathway. Associations were created using 800 k SNP data from Holstein and Jersey cattle. Purple bars represent the null hypothesis distribution. SNP sets were randomised from the 200 kb region spanning 67 genes. The vertical red line is the experimental result (e.g. the observed proportion of SNP in that pathway), while the green line is the P ≤ 0.05 significance threshold for the pathway from the permutation test, for **a)** fat kg, **b)** milk volume, **c)** protein kg, **d)** fat percentage, **e)** protein percentage. Click here for file

Additional file 6: Figure S4 Permutation tests for SNP within the prolactin signalling pathway. Purple bars represent the null hypothesis distribution. SNP sets were randomised from the 200 kb region spanning 27 genes. The vertical red line is the experimental result (e.g. the observed proportion of SNP in that pathway), while the green line is the P ≤ 0.05 significance threshold for the pathway from the permutation test, for **a)** fat kg, **b)** milk volume, **c)** protein kg, **d)** fat percentage, **e)** protein percentage. Click here for file

Additional file 7: Figure S5 Permutation tests for SNP within the involution pathway. Purple bars represent the null hypothesis distribution. SNP sets were randomised from the 200 kb region spanning 40 genes. The vertical red line is the experimental result (e.g. the observed proportion of SNP in that pathway), while the green line is the P ≤ 0.05 significance threshold for the pathway from the permutation test, for **a)** fat kg, **b)** milk volume, **c)** protein kg, **d)** fat percentage, **e)** protein percentage. Click here for file

Additional file 8: Table S3 Gene abbreviations. Gene families are represented in bold. Click here for file

Additional file 9: Figure S6 Control permutations with major QTL regions removed. Description: Histograms show **a)** mammary development with BTA14 removed and **b)** involution with BTA20 removed, both for protein percentage. Red lines represent the significance of the pathway. Green lines show the P value cut-off. Click here for file
