# Supplementary figures and images for: Genetic variants in mammary development, prolactin signalling and involution pathways explain considerable variation in bovine milk production and milk composition
Source: Genet Sel Evol. 2014 Apr 29;46(1):29. doi: 10.1186/1297-9686-46-29 (PMC4036308; doi:10.1186/1297-9686-46-29)

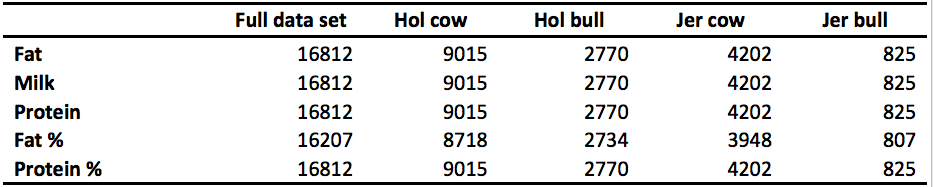

Supplement: Additional file 1: Table S1a — Number of phenotypes for production traits. Sample sizes adapted from [42]. Table S1b. The minimum and maximum phenotypes for production traits in dairy cattle. Phenotypes are expressed in standard deviations, with a mean of zero within each breed. Adapted from [42]. [file 1297-9686-46-29-S1.zip › 6351461661065383_add1a.png]

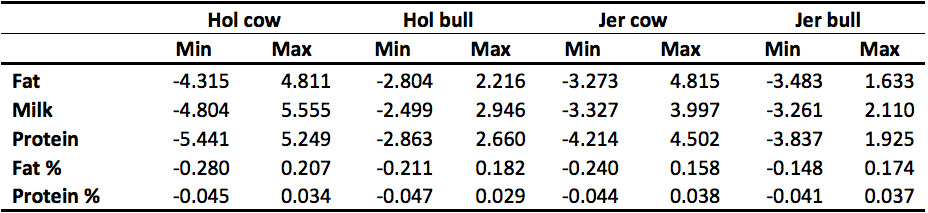

Supplement: Additional file 1: Table S1a — Number of phenotypes for production traits. Sample sizes adapted from [42]. Table S1b. The minimum and maximum phenotypes for production traits in dairy cattle. Phenotypes are expressed in standard deviations, with a mean of zero within each breed. Adapted from [42]. [file 1297-9686-46-29-S1.zip › 6351461661065383_add1b.png]

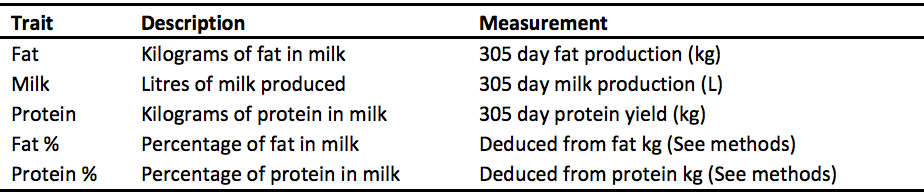

Supplement: Additional file 2: Table S2 — Description and measurement of milk production traits. All non-production traits are expressed as a percentage of the standard deviation from the phenotypic mean. [file 1297-9686-46-29-S2.png]

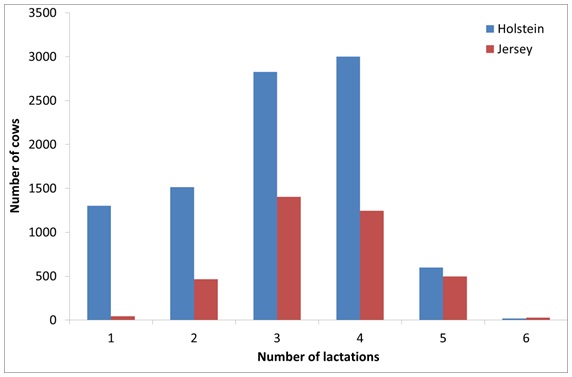

Supplement: Additional file 3: Figure S1a — Distribution of number of lactations for cows. X-axis is labelled with the mid-point of each bin. Figure S1b. Distribution of number of daughters per bull. X-axis is labelled with the mid-point of each bin. [file 1297-9686-46-29-S3.zip › 6351461661065383_add3a.jpeg]

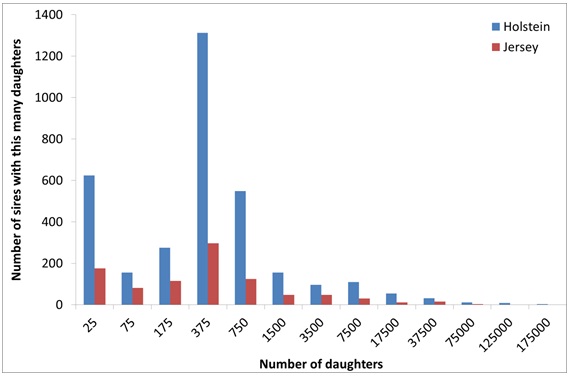

Supplement: Additional file 3: Figure S1a — Distribution of number of lactations for cows. X-axis is labelled with the mid-point of each bin. Figure S1b. Distribution of number of daughters per bull. X-axis is labelled with the mid-point of each bin. [file 1297-9686-46-29-S3.zip › 6351461661065383_add3b.jpeg]

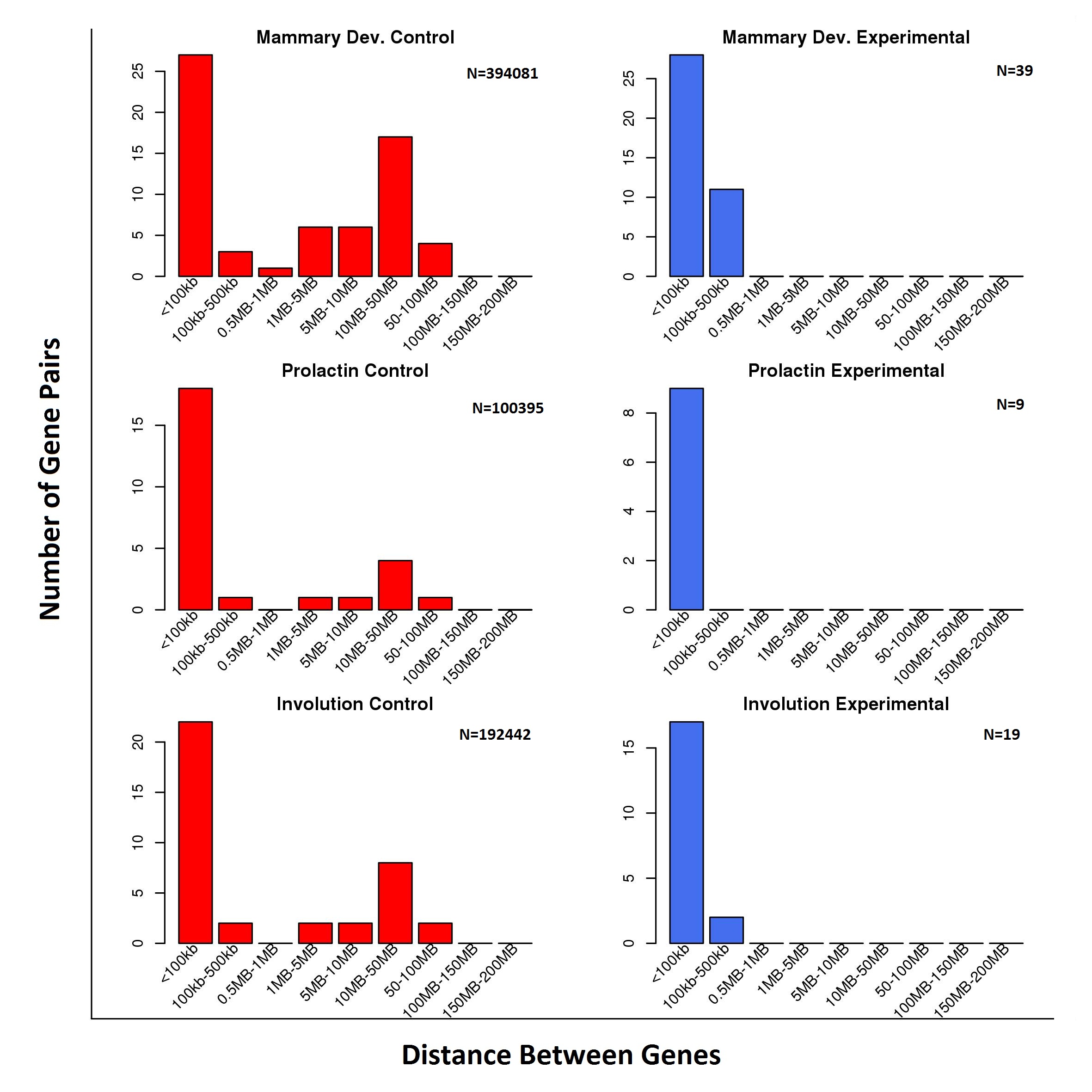

Supplement: Additional file 4: Figure S2 — Distance between genes in lactation pathways and control permutations. Control plots are scaled to represent 10 000 replicates of randomly selected genes of size equivalent to the experimental pathway. [file 1297-9686-46-29-S4.jpeg]

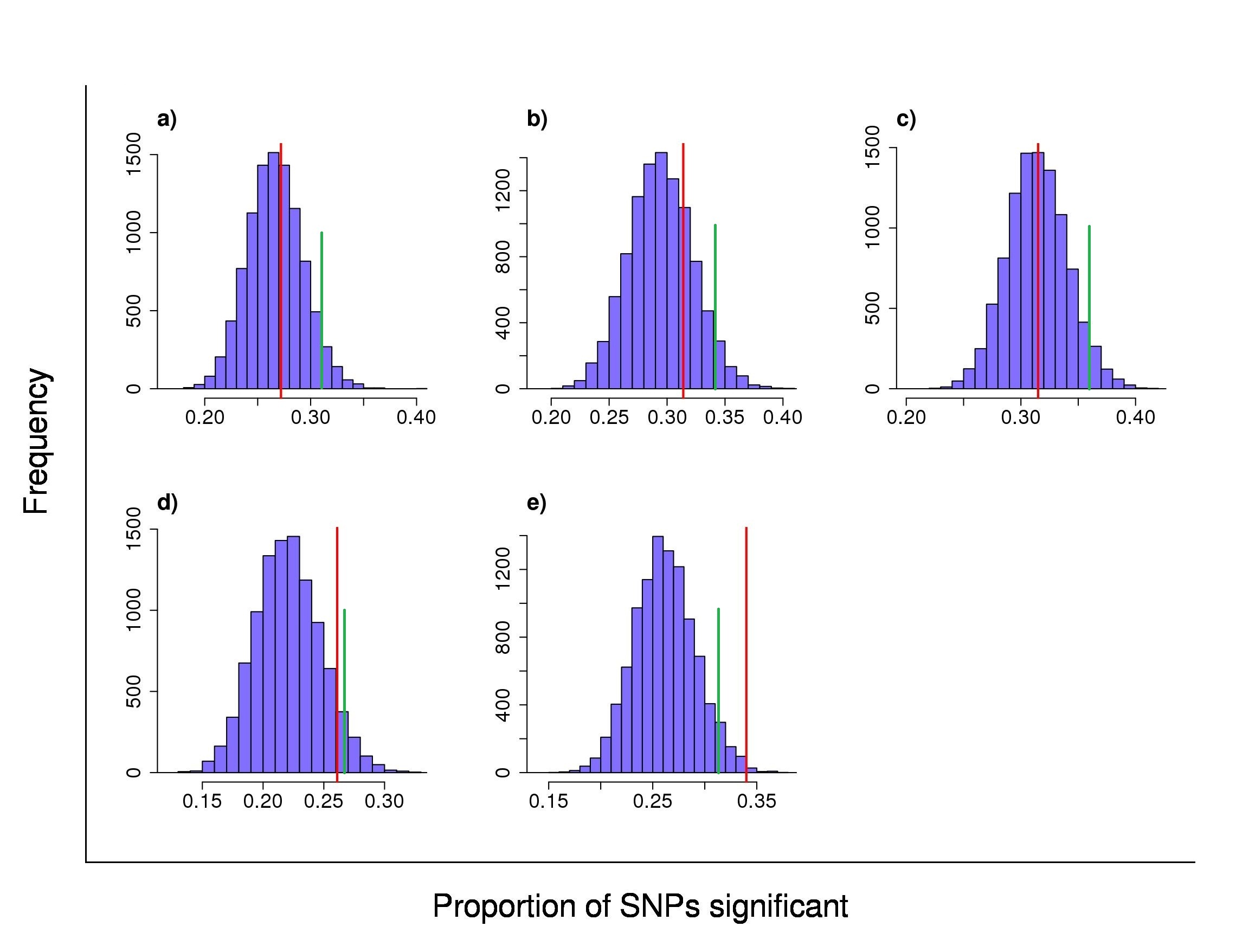

Supplement: Additional file 5: Figure S3 — Permutation tests for SNP within the mammary development pathway. Associations were created using 800 k SNP data from Holstein and Jersey cattle. Purple bars represent the null hypothesis distribution. SNP sets were randomised from the 200 kb region spanning 67 genes. The vertical red line is the experimental result (e.g. the observed proportion of SNP in that pathway), while the green line is the P ≤ 0.05 significance threshold for the pathway from the permutation test, for a) fat kg, b) milk volume, c) protein kg, d) fat percentage, e) protein percentage. [file 1297-9686-46-29-S5.jpeg]

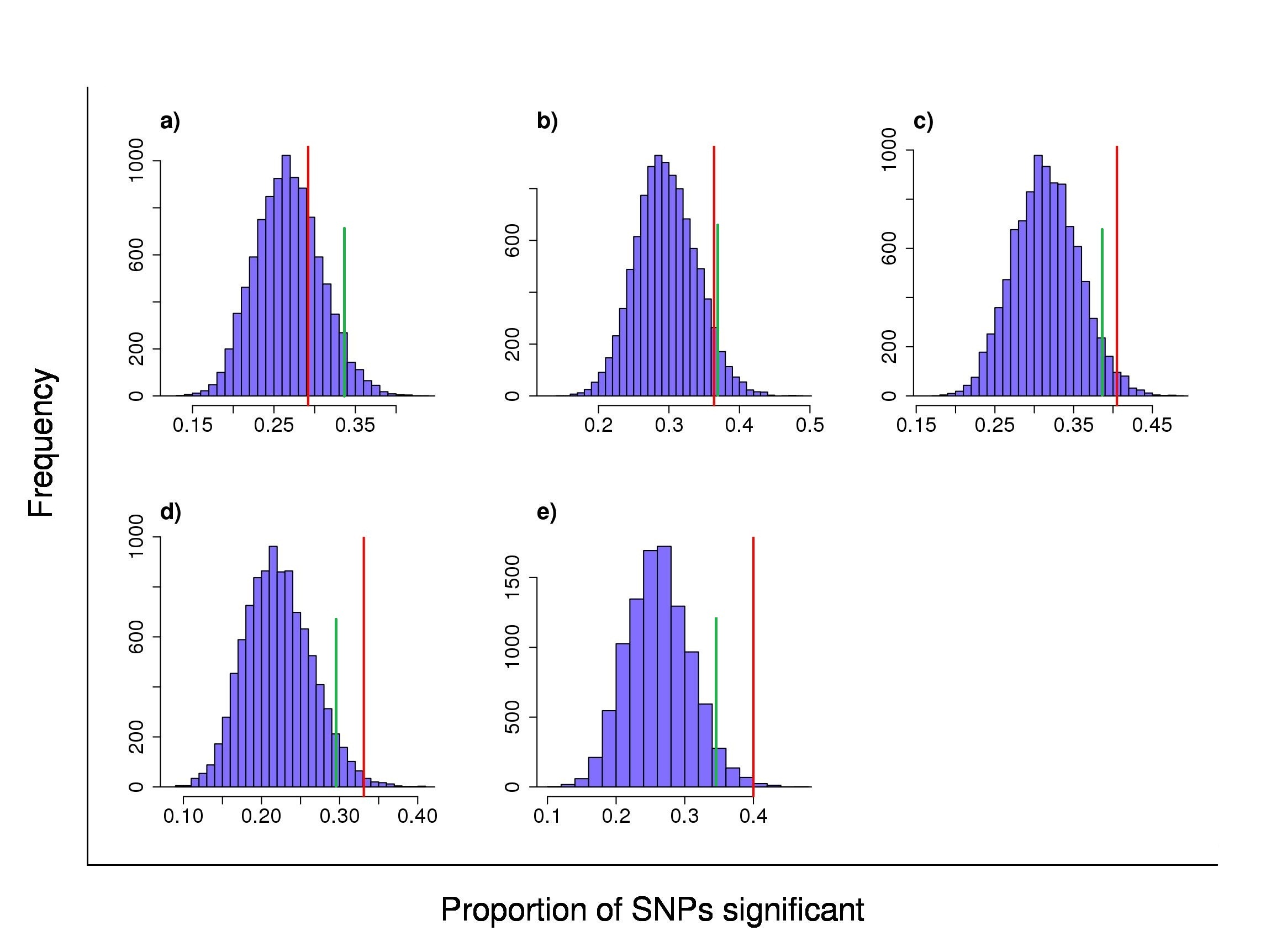

Supplement: Additional file 6: Figure S4 — Permutation tests for SNP within the prolactin signalling pathway. Purple bars represent the null hypothesis distribution. SNP sets were randomised from the 200 kb region spanning 27 genes. The vertical red line is the experimental result (e.g. the observed proportion of SNP in that pathway), while the green line is the P ≤ 0.05 significance threshold for the pathway from the permutation test, for a) fat kg, b) milk volume, c) protein kg, d) fat percentage, e) protein percentage. [file 1297-9686-46-29-S6.jpeg]

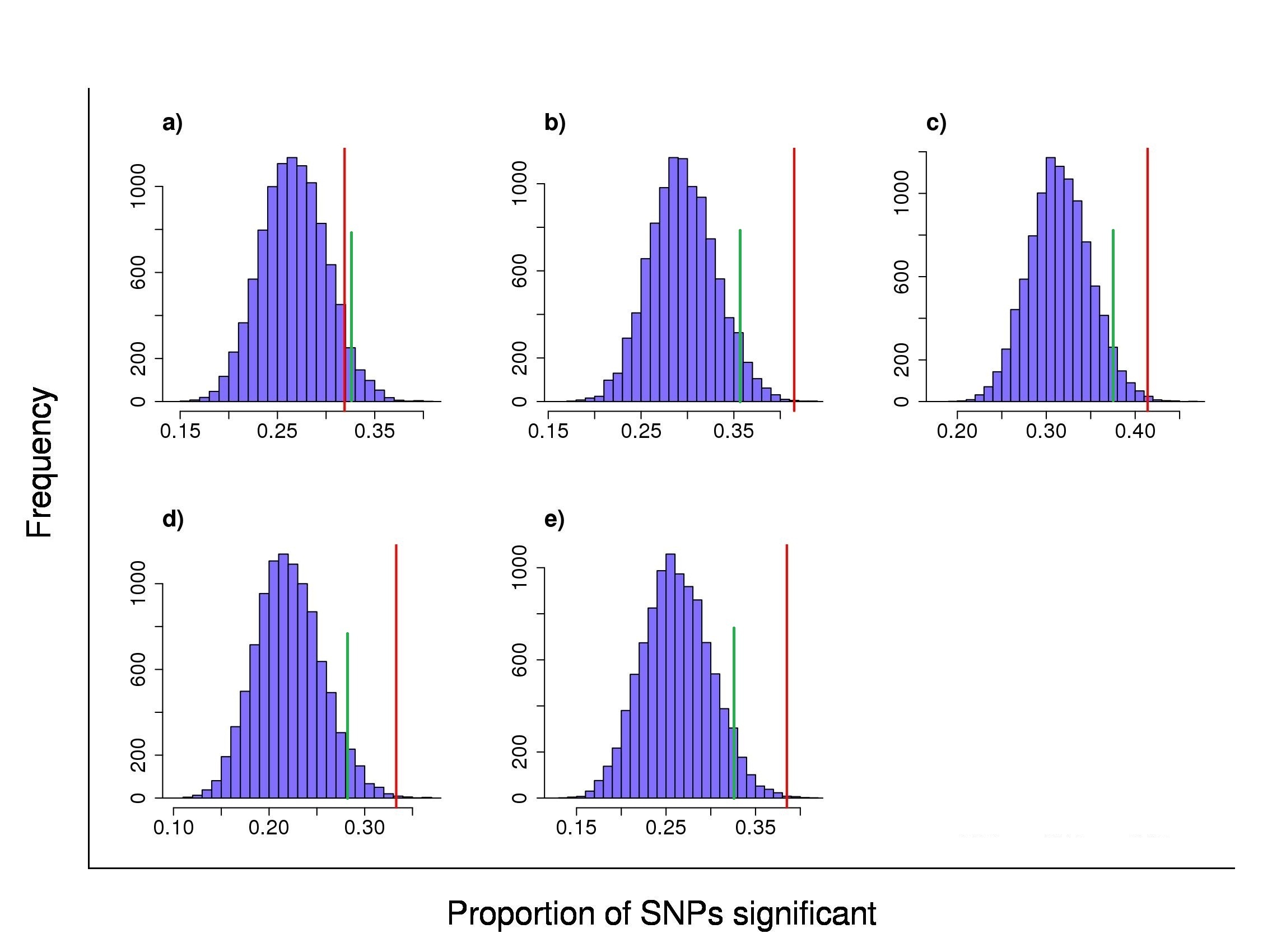

Supplement: Additional file 7: Figure S5 — Permutation tests for SNP within the involution pathway. Purple bars represent the null hypothesis distribution. SNP sets were randomised from the 200 kb region spanning 40 genes. The vertical red line is the experimental result (e.g. the observed proportion of SNP in that pathway), while the green line is the P ≤ 0.05 significance threshold for the pathway from the permutation test, for a) fat kg, b) milk volume, c) protein kg, d) fat percentage, e) protein percentage. [file 1297-9686-46-29-S7.jpeg]

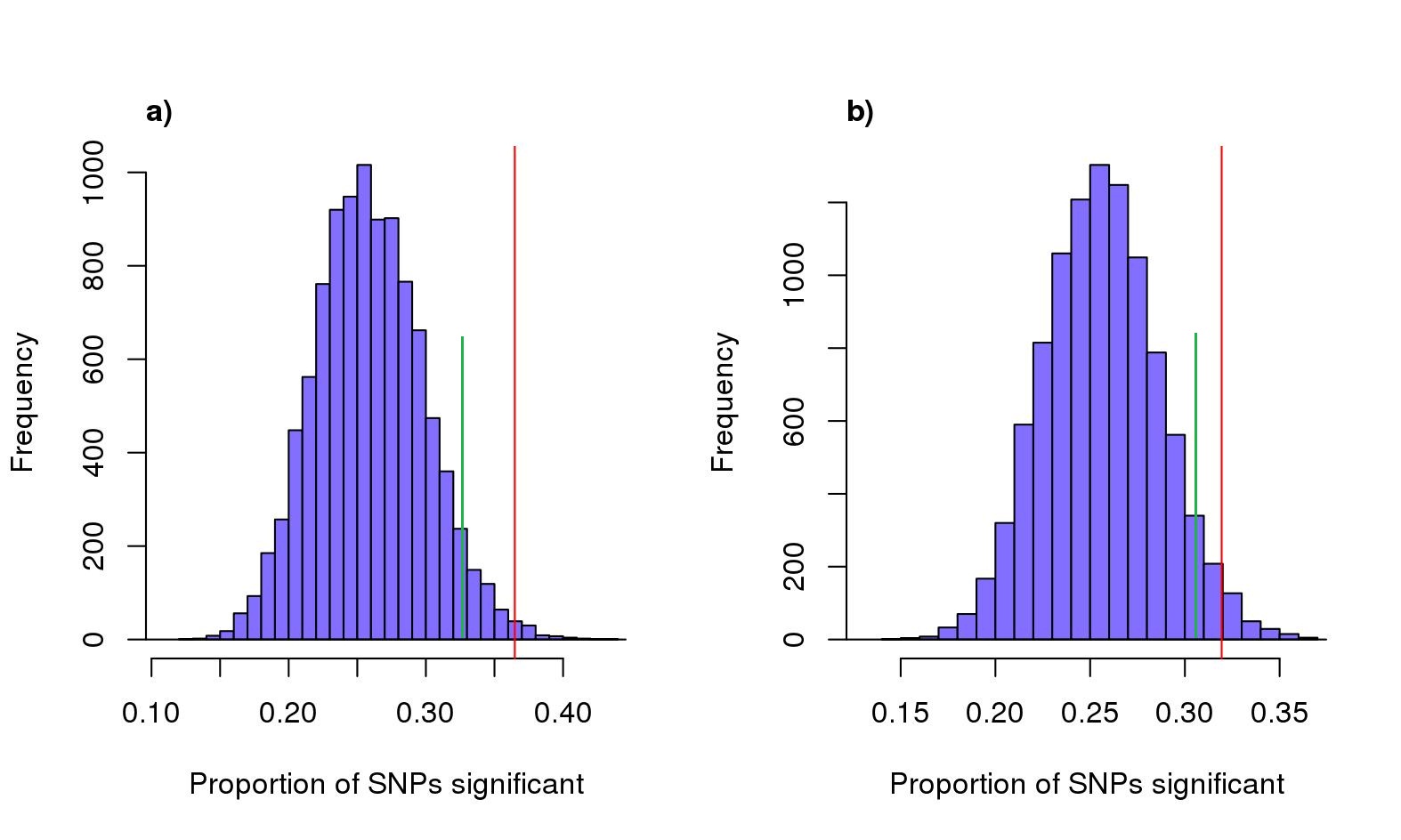

Supplement: Additional file 9: Figure S6 — Control permutations with major QTL regions removed. Description: Histograms show a) mammary development with BTA14 removed and b) involution with BTA20 removed, both for protein percentage. Red lines represent the significance of the pathway. Green lines show the P value cut-off. [file 1297-9686-46-29-S9.jpeg]
