# Supplementary material for: Genetic variants in mammary development, prolactin signalling and involution pathways explain considerable variation in bovine milk production and milk composition
Source: Genet Sel Evol. 2014 Apr 29;46(1):29. doi: 10.1186/1297-9686-46-29 (PMC4036308; doi:10.1186/1297-9686-46-29)
Supplement: Additional file 8: Table S3 — Gene abbreviations. Gene families are represented in bold. [file 1297-9686-46-29-S8.docx]

| **Gene** | **Description** |
| --- | --- |
| ADAM17 | ADAM metallopeptidase domain 17 |
| **AKT** | v-akt murine thymoma viral oncogene homolog |
| AREGB | Amphiregulin precursor |
| ATF4 | Activating transcription factor 4 (tax-responsive enhancer element B67) |
| BAK1 | BCL-2 homologous antagonist/killer |
| BAX | BCL2-associated X protein |
| BCL2L1 | B-cell CLL/lymphoma 2 Like |
| BMP4 | Bone morphogenetic protein 4 |
| BMPR1A | Bone morphogenetic protein Receptor 1A |
| CASP3 | Caspase-3 |
| CCND1 | G1/S-specific cyclin-D1 |
| **CEBP** | CCAAT/enhancer binding protein (C/EBP) |
| CISH | Cytokine-inducible SH2-containing protein |
| CSN1S1 | Casein alpha s1 |
| CSN2 | Casein beta |
| **CTNNA** | Catenin (cadherin-associated protein), alpha |
| DGAT1 | Diacylglycerol O-acyltransferase 1 |
| DKK1 | Dickkopf-related protein 1 |
| E2F1 | E2F transcription factor 1 |
| EDAR | Ectodysplasin A receptor |
| EGF | Epidermal growth factor |
| EGFR | Epidermal growth factor receptor |
| ELF5 | ETS-related transcription factor Elf-5 |
| ERBB4 | v-erb-b2 avian erythroblastic leukemia viral oncogene homolog |
| ESR1 | Estrogen receptor 1 |
| **FGF** | Fibroblast growth factor |
| FGFR1 | Fibroblast growth factor receptor 1 precursor |
| FOXO3 | Forkhead box protein O3 |
| GAL | Galanin prepropeptide |
| GATA3 | Trans-acting T-cell-specific transcription factor GATA-3 |
| GH1 | Growth hormone 1 |
| GHR | Growth hormone receptor |
| GLI | GLI family zinc finger |
| **IGF** | Insulin-like growth factor |
| IGF1R | Insulin-like growth factor 1 receptor |
| IGFBP5 | Insulin-like growth factor-binding protein |
| **IL** | Interleukin |
| IL6ST | Interleukin 6 signal transducer (gp130, oncostatin M receptor) |
| IRF1 | Interferon regulatory factor 1 |
| **IRS** | Insulin receptor substrate |
| **JAK** | Janus kinase |
| LEF1 | Lymphoid enhancer-binding factor 1 |
| LIF | Leukemia inhibitory factor |
| LIFR | Leukemia inhibitory factor receptor |
| MFGE8 | Lactadherin |
| **MMP** | Matrix metalloproteinase |
| **MSX** | MSH Homeobox |
| MYC | v-myc myelocytomatosis viral oncogene homolog (avian) |
| NR3C1 | Nuclear receptor subfamily 3, group C, member 1 (glucocorticoid receptor) |
| **NRG** | Neuregulin |
| NTN1 | Netrin 1 |
| OSM | Oncostatin M |
| OSMR | Oncostatin M Receptor |
| PCBD1 | Pterin-4 alpha-carbinolamine dehydratase/dimerization cofactor of HNF1A (TCF1) |
| PGR | Progesterone receptor |
| PRL | prolactin |
| PRLR | prolactin receptor long form precursor |
| PTEN | phosphatase and tensin homolog |
| PTH | Parathyroid hormone |
| PTH1R | Parathyroid hormone 1 receptor |
| PTHLH | Parathyroid hormone-like hormone |
| PTK2 | Protein tyrosine kinase 2 |
| RAF1 | v-raf-1 murine leukemia viral oncogene homolog 1 |
| RELN | Reelin |
| SFRP4 | Secreted frizzled-related protein 4 precursor |
| SIRPA | Tyrosine-protein phosphatase non-receptor type substrate 1 precursor |
| SLIT2 | Slit homolog 2 protein precursor |
| **SOCS** | Suppressor of cytokine signaling |
| **STAT** | Signal transducer and activator of transcription |
| **TBX** | T-box transcription factor TBX |
| **TCF** | Transcription factor E2-alpha |
| **TGF** | Transforming growth factor |
| **TGFBR** | TGF-beta receptor type |
| TIMP3 | Metalloproteinase inhibitor 3 precursor |
| TNFRSF11A | Tumor necrosis factor receptor superfamily, member 11a, NFKB activator |
| TNFSF11 | Tumor necrosis factor ligand superfamily member 11 |
| TP53 | Tumor protein p53 |
| WAP | Whey acidic protein |
| **WNT** | Wingless-type MMTV integration site family, member |
